# Enhanced rare disease mapping for phenome-wide genetic association in the UK Biobank

**DOI:** 10.1186/s13073-022-01094-y

**Published:** 2022-08-09

**Authors:** Matthew T. Patrick, Redina Bardhi, Wei Zhou, James T. Elder, Johann E. Gudjonsson, Lam C. Tsoi

**Affiliations:** 1 Department of Dermatology, University of Michigan Medical School, Ann Arbor, MI USA; 2 School of Medicine, Wayne State University, Detroit, MI USA; 3 Analytic and Translational Genetics Unit, Massachusetts General Hospital, Boston, MA USA; 4 Program in Medical and Population Genetics, Broad Institute of Harvard and MIT, Cambridge, MA USA; 5 Stanley Center for Psychiatric Research, Broad Institute of Harvard and MIT, Cambridge, MA USA; 6 Department of Biostatistics, Center for Statistical Genetics, University of Michigan, Ann Arbor, MI USA; 7 Department of Computational Medicine & Bioinformatics, University of Michigan, Ann Arbor, MI USA

**Keywords:** Rare disease, UK Biobank, Genetic associations, Phenotyping, Demographics

## Abstract

**Background:**

Rare diseases collectively affect up to 10% of the population, but often lack effective treatment, and typically little is known about their pathophysiology. Major challenges include suboptimal phenotype mapping and limited statistical power. Population biobanks, such as the UK Biobank, recruit many individuals who can be affected by rare diseases; however, investigation into their utility for rare disease research remains limited. We hypothesized the UK Biobank can be used as a unique population assay for rare diseases in the general population.

**Methods:**

We constructed a consensus mapping between ICD-10 codes and ORPHA codes for rare diseases, then identified individuals with each rare condition in the UK Biobank, and investigated their age at recruitment, sex bias, and comorbidity distributions. Using exome sequencing data from 167,246 individuals of European ancestry, we performed genetic association controlling for case/control imbalance (SAIGE) to identify potential rare pathogenic variants for each disease.

**Results:**

Using our mapping approach, we identified and characterized 420 rare diseases affecting 23,575 individuals in the UK Biobank. Significant genetic associations included *JAK2* V617F for immune thrombocytopenic purpura (*p = 1.24 × 10*^*−13*^) and a novel *CALR* loss of function variant for essential thrombocythemia (*p = 1.59 × 10*^*−13*^). We constructed an interactive resource highlighting demographic information (http://www-personal.umich.edu/~mattpat/rareDiseases.html) and demonstrate transferability by applying our mapping to a medical claims database.

**Conclusions:**

Enhanced disease mapping and increased power from population biobanks can elucidate the demographics and genetic associations for rare diseases.

**Supplementary Information:**

The online version contains supplementary material available at 10.1186/s13073-022-01094-y.

## Background

Rare diseases account for a high proportion of hospital visits [1, 2] and can severely reduce quality of life [3, 4]. A recent study [1] indicates ~10% of hospital discharges were patients who had a rare disease. Many rare diseases have a substantial impact on mortality [2, 5] and are associated with severe physical and mental disability [6]. Legislation has been introduced (e.g., the Rare Diseases Act and the Orphan Drug Act in the USA [7]) to encourage research and drug development for rare diseases, yet < 5% have an FDA approved treatment [5], and available treatments are often highly expensive [8] or inaccessible [9]. Delays in diagnosis are commonplace [3] and patients reported challenges in accessing specialists with experience of their condition [4]. Inference from Orphanet suggests 80% of rare diseases are genetic, yet disease-associated genes have been identified for < 40% of rare diseases [5]. A clearer understanding of rare disease pathogenesis is needed, to develop new treatments and reduce the burden on society.

Addressing challenges in healthcare for rare diseases is challenging due to the vast number and heterogeneity of rare conditions, as well as limited resources available to study each disease. There are believed to be > 10,000 rare diseases [10], with estimates varying due to divergent definitions of rarity [5] and lack of consensus over which diseases constitute distinct entities [11]. It can be difficult to collect sufficient samples for statistical significance, due to low prevalence [12], and clinical trials for rare diseases typically include fewer participants [13]. There are ongoing attempts to establish biobanks for specific rare diseases, such as the EuroBioBank network [14], which provide access to samples for research, and information to guide clinical trials. However, biobanks are not available for every disease, and they cannot be used to understand demographics of rare diseases in the general population.

Population-based biobanks, such as the UK Biobank (UKB) [15] and All of Us research program [16], provide extensive information (both phenotypes and genotypes) on individuals with a wide range of (rare and common) diseases, reflecting the general population. The overall prevalence of having at least one rare disease has been estimated at > 3.5% in Europeans [17], which is higher than many common diseases, such as psoriasis [18] and glaucoma [19]; some sources suggest as many as 1 in 10 Americans are living with a rare disease [4, 10]. One difficulty in harnessing population biobanks is their diagnosis coding systems (e.g., the 10th revision of the International Statistical Classification of Diseases and Related Health Problems, ICD-10) are not specifically designed for rare diseases. ICD-10 codes are typically broader than those from rare disease nomenclatures (such as Orphanet [20]), meaning that while it is possible to map from ORPHA codes (in Orphanet) to ICD-10 for billing purposes, individuals annotated with those ICD-10 codes do not necessarily have a rare disease.

We hypothesize that applying enhanced phenotype mapping to the large sample sizes of population biobanks can provide valuable information regarding the demographics of rare diseases and could help advance the study of previously underserved conditions. We perform a consensus mapping of ICD-10 to ORPHA codes, to identify which ICD-10 codes reliably indicate rare diseases, then use it to catalog and explore the data available on rare diseases through the UKB. To facilitate the dissemination of this information, we have created an online resource (http://www-personal.umich.edu/~mattpat/rareDiseases.html) which will enable researchers to investigate specific rare diseases in the UKB and other systems that rely on ICD-10 codes. Furthermore, our association studies highlight significant genetic-disease associations, shedding light on the importance of refined phenotype mapping.

## Methods

### Mapping ICD-10 to ORPHA codes

From the ICD-10 mapping provided by Orphanet (which does not require the ICD-10 codes to be as specific as their corresponding ORPHA codes), we conducted refined mapping such that an individual annotated with an ICD-10 code can be expected to have the rare disease indicated by the corresponding ORPHA code (Supplementary Figure 1). For example, Orphanet maps ORPHA:314 (Leiner’s disease) to L21.1 (seborrheic infantile dermatitis); however, seborrheic dermatitis is in general common among newborns [21], so this ICD-10 code is excluded by our approach. We thus required the ORPHA code selected for each ICD-10 code to be as specific as possible (i.e., provide the most narrow and precise description), but no more specific than the ICD-10 code, such that we are able to reliably map ICD-10 codes to ORPHA codes representing specific rare diseases. No parent ICD-10 codes of the mapped ICD-10 codes are used in our mapping.

Clinical and demographic data, from 502,493 individuals in the UKB, was downloaded on 24 August 2020, with diagnoses annotated by ICD-10 codes. We started with 2,044 ICD-10 codes from the UKB mapped to 6762 ORPHA codes by Orphanet and applied a consensus approach to identify the subset of mappings that meet our criteria, selecting a single ORPHA code for each ICD-10 code (Additional file 1: Table S1). It should be noted that multiple ICD-10 codes can map to the same ORPHA code if the ICD-10 codes represent subtypes of the rare disease; for example, multisystemic (C96.0), unisystemic (C96.5), and unifocal (C96.6) Langerhans cell histiocytoses are all mapped to ORPHA:389 (Langerhans cell histiocytosis). Putative ICD-10/ORPHA pairs were assessed independently (by RB and MTP), and then the assessments were compared together. As the third voter in our consensus, we employed the results of a previous study mapping a subset of the Australian modification of ICD-10 (ICD-10-AM) to ORPHA codes [1]. Where assessments differed, further investigation was conducted until a joint decision could be reached. The end result is a set of ICD-10 codes for each ORPHA code that can be reliably mapped, allowing rare diseases to be identified in the UKB and other resources that use ICD-10 codes.

### UKB data analysis

We extracted the subset of individuals with ICD-10 codes from primary or secondary diagnoses corresponding to each ORPHA code in our mapping for rare diseases. Disease prevalence was estimated by dividing the number of individuals with ICD-10 codes mapping to those ORPHA codes by the total number of individuals in the UKB (of all ancestries). We compared the estimated prevalence for each rare disease with criteria for considering a disease as rare: < 1 in 2,000 people in Europe (< 0.05%) or < 200,000 people in the USA (< 0.06%) [5]. Statistics describing the proportion of males, age at recruitment, and comorbidities (collected from the entire study period) were compiled for each disease from the available data in the UKB. Diseases were grouped based on the different chapters in the ICD-10 coding system, splitting off “Other Immune” from Chapter III for D80-D89, then combining Chapters V and VI as neurological diseases, VII and VIII as Eye/Ear, chapters XV and XVI as pregnancy/childbirth, and XVIII, XX, XXI, and XXII as Miscellaneous. Access to data from the UK Biobank (https://www.ukbiobank.ac.uk/learn-more-about-uk-biobank/about-us/ethics) was obtained through a material transfer agreement which falls within the UK Biobank’s generic Research Tissue Bank (RTB) approval from the NHS North West REC.

### Comorbidities

We investigated the prevalence of different groups of comorbidities among individuals with different groups of rare diseases using the groups we identified from the ICD-10 codes. We also compared the enrichment of these groups of comorbidities against two previous studies (one for common diseases [22] and one for Mendelian diseases [23]) using Fisher exact tests.

### Exome sequencing analysis

We performed genetic association analysis on 167,246 individuals of European ancestry from the UKB who self-reported as White British and were genetically confirmed by principal components analysis (UKB field 22,006), using exome sequencing data prepared with the Original Quality Functionally Equivalent (OQFE) protocol. We restricted the genetic analysis to individuals of European ancestry because they constitute the vast majority of the UK Biobank and imbalanced cases/controls for rare diseases could otherwise lead to false positives for variants associated with ancestry. Variant- and gene-level association tests were conducted using SAIGE/SAIGE-GENE [24, 25], a generalized mixed model that provides accurate results in situations where there are far fewer cases than controls and applies a sparse relatedness matrix to account for population stratification. Variants were required to be rare (minor allele frequency, MAF ≤ 1%) in the full population but have a minor allele count of at least three among individuals with the rare disease and more than three overall, annotated as having a high or moderate impact by SnpEff [26] or a score ≥ 0.75 by REVEL [27], resulting in 33,981 variants across 162 rare diseases. Significant variant and gene associations from SAIGE were compared against the genes annotated for each disease by Orphanet and nine different variant effect predictors (CADD, FATHMM, LRTori, MetaSVM, MutationAssessor, MutationTaster Polyphen2 using HDIV or HVAR, PROVEAN and SIFT) annotated in dbNSFP v4.1 [28]. We then evaluated the pathogenicity of variants identified using the 2015 ACMG/AMP classification [29]. We also investigated loss of function variants predicted using SnpEff [26] by filtering the SAIGE associations to those variants. Significant rare disease associations were further filtered to highlight diseases which, in addition to being indicated by Orphanet as rare in Europe (< 1 in 2000 people) are indicated by NIH’s Genetic and Rare Diseases Information Center (GARD) as being rare in the USA (< 200,000 people).

### Confirmation using other data sources

To illustrate how our ICD-10/ORPHA mappings can facilitate rare disease research in additional datasets, we applied them to a nationwide database of medical claims from 39 million patients (Optum’s deidentified Clinformatics® Data Mart [30]) and compared the estimated prevalence of each disease against the UKB. Since a large proportion of diagnoses in Clinformatics® are coded using ICD-9, it is necessary to find equivalent ICD-9 codes for the ICD-10/ORPHA mappings. However, the ICD-9 coding system is less detailed than ICD-10, particularly for rare diseases. We therefore used the 2018 General Equivalence Mappings (GEMS) published by the Centers for Medicare and Medicaid Services [31] and excluded any diseases labeled as having approximate mappings. Although this limits the number of diseases that can be mapped, it allows for more accurate disease identification. Access to data from the Clinformatics® Data Mart (https://www.optum.com/content/dam/optum/resources/productSheets/Clinformatics_for_Data_Mart.pdf) was obtained through a data use agreement according to a license agreement at the University of Michigan Institute for Healthcare Policy and Innovation (IHPI).

### Statistical analysis

We applied Bonferroni adjustment to identify significant variants and gene level associations from SAIGE and SAIGE-gene, respectively. Pearson correlation and Mann-Whitney tests were used to assess the associations between age/gender and rare disease diagnoses. We also used Pearson correlation and test of proportions to compare the prevalence of rare diseases in Clinformatics® and the UK Biobank. In each case, we present both the effect size and *p*-value, as appropriate.

## Results

### Rare diseases identified

Table 1 provides some examples of our disease mapping, along with the number of individuals in the UKB with the rare disease and group (identified from the ICD-10 chapter) the disease belongs to (Additional file 1: Table S2 contains the full list). Using our consensus approach, we mapped 1,176 ICD-10 codes to 720 ORPHA codes. 23,575 individuals in the UKB (~5% prevalence) were found to have at least one of 420 specific rare diseases, with 2,602 individuals having more than one rare disease (~10% prevalence). It is interesting that individuals with a rare disease have higher susceptibility to other rare diseases, as this suggests some of the rare conditions may be related, either causally or by shared molecular mechanisms. This finding confirms a previous study in the USA [4], which revealed that ~13% of individuals with a rare condition have more than one.

**Table 1:** Sample sizes obtained for some of the rare disorders. For each of the rare diseases we identified in the UK Biobank, we provide the set of ICD-10 codes that map directly to the codes from Orphanet, such that the ORPHA code is no more specific that the ICD-10 codes. We then provide the number of individuals with that rare disease in the UK Biobank (UKB count) along with the group the disease belongs to, based on its ICD-10 chapter

Disease name	ORPHA code	ICD-10 code	UKB count	Group
Addison’s disease	85138	E27.1	242	Endocrine/metabolic
Waldenström macroglobulinemia	33226	C88.0	109	Neoplasms
Marfan syndrome	558	Q87.4	93	Congenital
Beta-thalassemia	848	D56.1	71	Blood
Autosomal dominant tubulointerstitial kidney	34149	Q61.5	41	Congenital
Congenital ptosis	91411	Q10.0	31	Congenital
Tetralogy of Fallot	3303	Q21.3	19	Congenital
Congenital renal artery stenosis	97598	Q27.1	7	Congenital
Autosomal dominant epidermolytic ichthyosis	312	Q80.3	<5	Congenital
Fragile X syndrome	908	Q99.2	<5	Congenital
Reye syndrome	3096	G93.7	<5	Neurological

**Fig. 1:**
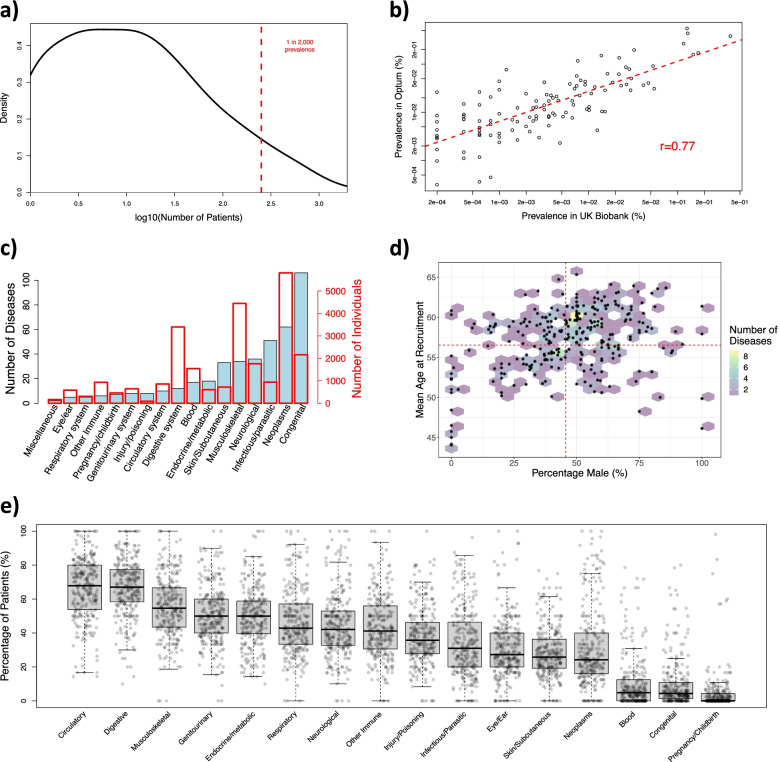
Rare diseases in the UK Biobank. **a** Density plot of the 420 rare diseases we identified in the UK Biobank by mapping ICD-10 codes to ORPHA codes. The *x*-axis shows the log10 number of individuals recorded as having each disease, while the *y*-axis shows the density of diseases with that number of individuals. The red dashed line indicates the (fewer than 1 in 2000) prevalence criterion for rare diseases in Europe. **b** Scatter plot comparing the prevalence of rare diseases in the UK Biobank and the Optum dataset. Each point represents a rare disease, and the dotted red line represents the linear regression between the prevalence in Optum and the UK Biobank. **c** The groups of rare diseases identified in the UK Biobank are shown as a bar plot, with the *y*-axis indicating the number of diseases in each group. Overlaid is a second bar plot (in red), with the *y*-axis indicating the number of individuals who have at least one disease in each group. **d** Hexagon/scatter plot showing the mean age at recruitment and proportion of males for each disease. The *x*-axis shows the percentage of males recorded as having each disease and the *y*-axis shows the mean age at recruitment. The hexagons show the density of diseases at each mean age and sex proportion, while the asterisks indicate the actual values for a particular disease. The red dashed line shows the overall mean age and sex proportion in the UK Biobank. **e** Box/scatter plot of comorbidities for rare diseases. The *y*-axis shows, for each rare disease, the percentage of individuals who have at least one comorbidity in each group (excluding the rare disease itself)

Figure 1a presents the distribution of the number of individuals who are recorded as having each disease, with the highest disease density occurring for 5.4 individuals (1 in 100,000 estimated prevalence). A similarly high density is observed for diseases with up to 30 individuals, and density decreases as the number of individuals increases. The vast majority of diseases (indicated as rare in Orphanet) also meet the criteria for being rare in the UKB, suggesting our mapping approach has successfully identified the rare diseases. However, 24 out of the 420 diseases (6%) have an estimated prevalence greater than 1 in 2000 (indicated by the dashed red line), meaning they are not rare by the European definition. Furthermore, 21 of these diseases do not meet the US criterion (affecting fewer than 200,000 people) when extrapolating to the population of the USA. This is important to consider with respect to the demographics of rare diseases in the UKB.

The total number of rare disease individuals in the UKB differs depending on whether diseases with >1/2,000 prevalence are included (23,575 individuals, 5% of the total) or excluded (10,635 individuals, 2% of the total). Both estimates are within the range of rare disease prevalence previously reported [4, 10], and for the remainder of our analysis, we include all 420 diseases, using Orphanet as the deciding factor over whether a disease should be considered rare. It is also worth noting our approach is conservative, since it only includes rare diseases that can be reliably mapped from ICD-10 to ORPHA. As many rare diseases are not precisely defined within ICD-10, so are excluded by our mapping approach, we should expect the overall prevalence of rare diseases to be higher than our reported number. However, it is also possible due to inaccuracies in diagnosis that some individuals annotated with a particular ICD-10 code might not have the disease it refers to.

### Confirmation of applicability to Optum’s deidentified Clinformatics® Data Mart

By converting the ICD-10 codes from our consensus mapping to non-approximate ICD-9 codes, we created ICD-9 to ORPHA mappings for 114 rare diseases (Additional file 2: Table S3). We applied our ICD-9/10 mappings to Clinformatics® and compared the prevalence of individuals with each rare disease to the UKB (Fig. 1b). The prevalence of rare diseases in Clinformatics® and the UKB is highly correlated (*r* = 0.77, *p* = 2.2 × 10^−23^). Interestingly, celiac artery compression syndrome had substantially higher prevalence in Clinformatics® (0.044%) compared to the UKB (0.001%), as did burning mouth syndrome (Clinformatics®: 0.117%, UKB: 0.008%). However, generally rare diseases had higher prevalence in Clinformatics®, with only 11 having higher prevalence in the UKB. We further compared the prevalence estimated from the UKB against those reported in Orphanet and found that of the 186 rare diseases from our mapping that have prevalence information in Orphanet, 165 (89%) are within one order of magnitude difference; 66 (35%) have higher prevalence in the UKB, and 48 (26%) have lower prevalence in the UKB, compared with the range reported by Orphanet.

The relatively high recruitment age in the UKB might be expected to result in more rare diseases arising or being identified. Polymyalgia rheumatica is more common among older adults and does have a slightly higher prevalence in the UKB (0.39%) than Clinformatics® (0.38%). However, trigeminal neuralgia is also more common in older adults and has more than twice the prevalence in Clinformatics® (0.27%) than the UKB (0.12%), fold change (FC = 2.29, *p* = 9.03 × 10^−208^, test of proportions). Both Clinformatics® and the UKB may be subject to selection biases. In addition to variation between the UK and USA, there are also data collection differences: Clinformatics® claims are enriched for individuals in contact with a health system, while the UKB is community focused but may be biased towards healthy volunteers [32].

### Quantifying groups of rare diseases

Figure 1c presents the number of rare diseases identified in the UKB for each non-overlapping group defined from ICD-10 chapters (congenital, neoplasms and infectious/parasitic rare diseases are the most numerous); although individuals can have diseases from multiple groups, each disease belongs to exactly one group. Overlaid (in red) are the number of individuals with at least one disease from each group (neoplasms, musculoskeletal and digestive system diseases are the most common). Orphanet also provides an overlapping set of categories (Additional file 2: Figure S1); each disease is associated with multiple categories, with genetic, neurological, and hematological categories among the most frequent.

### Demographics in the UKB

From the UKB, we extracted the age at recruitment, gender, and comorbidities for individuals who have each disease. Individuals with a rare disease had slightly higher median age (60) than those without (58), Mann-Whitney *p = 9.0 × 10*^*−314*^ FC = 1.03; individuals with more than one rare disease also had a higher median age (61) than those with only one rare disease (60), Mann-Whitney *p = 6.5 × 10*^*−6*^ FC = 1.02 (some individuals with an ICD-10 code might not have the disease due to inaccuracies in diagnosis). Figure 1d and Additional file 2: Figure S2 show a modest (positive) correlation (*r* = 0.32, *p = 4.8 × 10*^*−8*^) between male proportion and median age at recruitment for different rare diseases, with male dominated diseases having higher age at recruitment. Some male-biased diseases have a low median recruitment age, including cocaine intoxication and Kaposi’s sarcoma (associated with HIV [33]). There are also female-biased diseases with older age, such as malignant tumor of fallopian tubes and vulvar intraepithelial neoplasia.

**Fig. 2:**
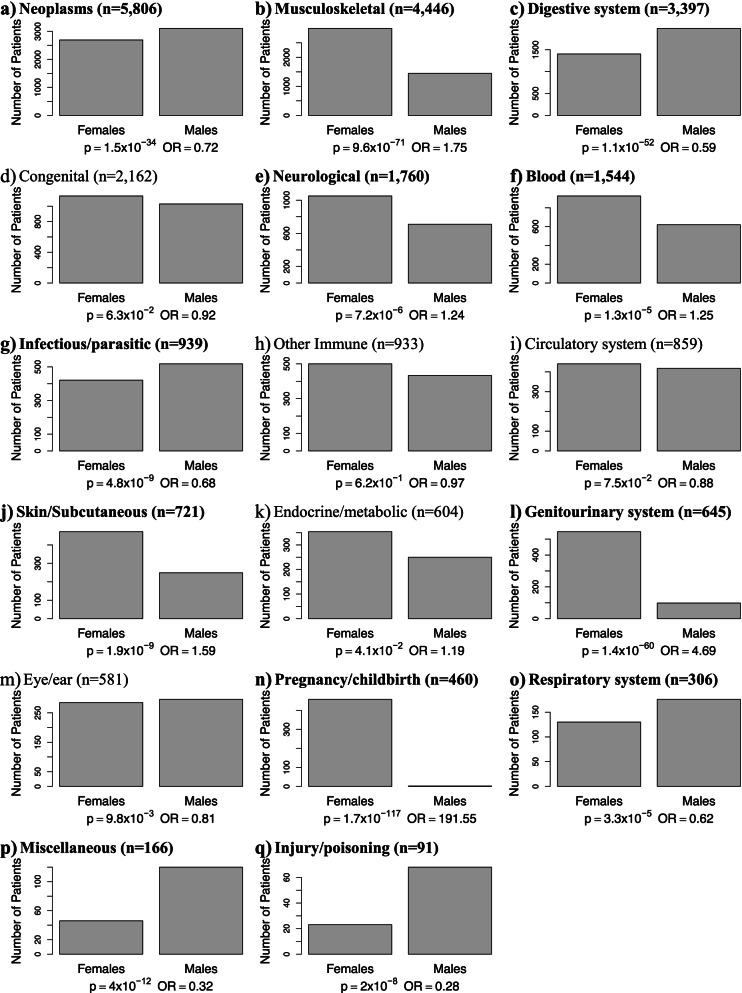
Sex of individuals with different groups of rare disease. Each bar plot presents the number of male and female individuals in the UK Biobank who have at least one rare disease from a particular group. Non-overlapping groups of rare diseases were identified from their corresponding ICD-10 chapters. For each group, we conducted a Fisher enrichment test, comparing the number of males and females in the group with the number of males and females in the UK Biobank overall; *p*-values and odds ratios are provided under each bar plot and the subtitles of groups with significant sex bias are indicated in bold font

Although rare diseases affected a similar proportion of males and females overall, approximating the general population, there are considerable differences in sex proportion between rare disease groups (Fig. 2 and Additional file 2: Figure S3). Neoplasms and digestive system rare diseases are more frequent among males, while musculoskeletal and skin/subcutaneous rare diseases are more frequent among females. Figure 1e and Additional file 2: Figure S4 present the comorbidities of rare diseases, determined by ICD-10 codes. The percentage of individuals with comorbidities in each group differs substantially between rare diseases, illustrating diversity in how rare diseases relate to common comorbidities and suggesting the broad impact of rare diseases. Additional file 2: Figure S5 shows the enrichment of comorbidities among individuals with different groups of rare diseases as a heatmap. Most groups were significantly enriched for comorbidities in the same group (i.e., on the diagonal), while individuals with rare blood or genitourinary disorders were more likely to have pregnancy/childbirth comorbidities, and individuals with rare respiratory system disorders were more likely to have infectious/parasitic comorbidities.

We compared our findings with those from a recent work [22] that studied comorbidities of common diseases in the UK Biobank (Additional file 2: Figure S6); having a comorbidity in the same category was generally less common for rare diseases than for common diseases, with the notable exception of rare respiratory system diseases, which have higher same-category and cross-category enrichment. As with our study (Additional file 2: Figure S5), the Dong et al. study reported significant comorbidities between common infectious and respiratory disorders, as well as between common respiratory and metabolic conditions. However, uniquely, we observed individuals with a rare form of neoplasm are more likely to have a blood disorder, whereas individuals who have rare blood disorders are not necessarily more likely to have neoplasms. Overall, the differences in enrichments across the disease categories between our study and the common disease study are minor, further supporting the accuracy of our analysis. We further compared our results with a study that investigated the association between Mendelian and complex genetic diseases [23], focusing on the 15 rare diseases from our mapping in the UK Biobank that it included. Interestingly, we identified 10 significant enrichments (Additional file 2: Figure S7) after Bonferroni correction, all of which were supported by the previous study. Although we did not observe any significantly enriched comorbidities between the Mendelian diseases, we expanded our analysis to all the rare diseases from our mapping and identified 222 significantly co-occurring rare diseases (Additional file 1: Table S4) and 389 significant complex disease comorbidities of rare diseases (Additional file 1: Table S5) after Bonferroni correction.

### Interactive web browser for rare diseases in the UKB

To help facilitate the use of our findings for future rare disease research, we created a website (http://www-personal.umich.edu/~mattpat/rareDiseases.html) which allows rare diseases within the UKB to be easily explored. Our website includes an interactive table with details of each rare disease (their name, ORPHA code, ICD-10 codes, number of individuals in the UKB, and disease group). When the user accesses a disease in the table, more information is provided in the tabs above, including the prevalence, age at onset, age at death, inheritance type, genes, and categories from Orphanet (where available), in addition to a table of phenotypes and their expected frequency. On different tabs are figures showing the age at recruitment and sex of individuals, relative to the general population, and the proportion of individuals with different comorbidities, with a link to more information regarding the disease from Orphanet. To ensure the anonymity of participants in the UKB, no age, sex, or comorbidity information is shown for diseases affecting fewer than five individuals.

### Exome sequencing analysis

Applying SAIGE/SAIGE-GENE to 33,981 variants across 162 diseases indicated as rare by Orphanet (< 1 in 2000 people) and using Bonferroni adjustment, we identified 19 significant variant-level associations and 20 significant gene-level associations. We further restricted to diseases indicated by NIH’s GARD as rare (< 200,000 people), resulting in 14 variant-level associations (Table 2) and 14 gene-level associations (Additional file 2: Table S6) significant for rare diseases. The full set of gene-level associations and variant level associations with false discovery rate (FDR) ≤ 0.05 are provided in Additional file 1: Table S7 and Additional file 1: Table S8, respectively. Six of the 14 variant-level associations (43%) had previously been reported in ClinVar.

**Table 2:** Significant variant-level associations. We applied SAIGE’s GLMM test with Bonferroni adjustment to identify significant variant associations for each rare disease and Bonferroni adjustment is applied. Genomic positions are provided in hg38 build and HGVS nomenclature. The ACMG/AMP classification for each association was determined through the use of Varsome and InterVar. Associations are specified as being reported if they have previously been indicated as pathogenic or likely pathogenic in ClinVar for that disease. Abbreviations are as follows: MAC, minor allele count; MAF, minor allele frequency. Although additional significant variant-level associations were identified for systemic lupus erythematosus and von Willebrand disease, these diseases were indicated as not being rare in the USA by NIH’s GARD. Furthermore, significant variant-level associations with interatrial communication, benign epithelial tumor salivary glands, and endophthalmitis were excluded because these diseases were not listed on NIH’s GARD, so it is difficult to confirm their rareness in the USA

Disease	ORPHA	Marker	ACMG/AMP classification	Reported?	***p***-value	Case MAC	Percent affected	Control MAF
Polycythemia vera	729	9:5073770_G/T (*JAK2*: missense) NC_000009.12:g.5073770G>T	Pathogenic (PS3/PS4)	Yes	1.32 × 10^−114^	51/370	47%	1.71 × 10^−04^
Chronic myeloproliferative disease	86830	9:5073770_G/T (*JAK2*: missense) NC_000009.12:g.5073770G>T	Pathogenic (PS3/PS4)	Yes	2.40 × 10^−67^	30/154	28%	2.33 × 10^−04^
Essential thrombocythemia	3318	9:5073770_G/T (*JAK2*: missense) NC_000009.12:g.5073770G>T	Pathogenic (PS3/PS4)	Yes	2.91 × 10^−42^	21/218	19%	2.60 × 10^−04^
Primary myelofibrosis	824	9:5073770_G/T (*JAK2*: missense) NC_000009.12:g.5073770G>T	Pathogenic (PS3/PS4)	Yes	5.30 × 10^−40^	16/52	15%	2.75 × 10^−04^
Immune thrombocytopenic purpura	3002	9:5073770_G/T (*JAK2*: missense) NC_000009.12:g.5073770G>T	Pathogenic (PS3/PS4)	No	2.63 × 10^−18^	11/368	10%	2.90 × 10^−04^
Chronic myelomonocytic leukemia	98823	17:76736877_G/A (*SRSF2*: missense) NC_000017.11:g.76736877G>A	Likely pathogenic (PS4/PM1)	No	1.19 × 10^−13^	4/32	27%	3.29 × 10^−05^
Essential thrombocythemia	3318	19:12943813_A/ATTGTC (*CALR*: frameshift variant) NC_000019.10:g.12943813_12943814insTTGTC	Pathogenic (PVS1/PS4)	No	2.82 × 10^−13^	5/218	50%	1.50 × 10^−05^
Beta-thalassemia	848	11:5226774_G/A (*HBB*: stop gained) NC_000011.10:g.5226774G>A	Pathogenic (PVS1/PS4)	Yes	3.46 × 10^−12^	3/12	33%	1.79 × 10^−05^
Congenital factor XI deficiency	329	4:186288589_T/G (*F11*: missense) NC_000004.12:g.186288589T>G	Pathogenic (PS4/PM1/PM2/PP2/PP3)	No	3.41 × 10^−11^	3/18	12%	6.58 × 10^−05^
B-cell chronic lymphocytic leukemia	67038	3:38141150_T/C (*MYD88*: stop lost) NC_000003.12:g.38141150T>C	Pathogenic (PS4/PM2/PM4/PP3/PP5)	Yes	2.42 × 10^−10^	4/490	57%	8.98 × 10^−06^
Acute panmyelosis with myelofibrosis	86843	9:5073770_G/T (*JAK2*: missense) NC_000009.12:g.5073770G>T	Pathogenic (PS3/PS4)	No	7.81 × 10^−10^	3/8	3%	3.14 × 10^−04^
Immune thrombocytopenic purpura	3002	16:83907050_G/A (*MLYCD*: missense) NC_000016.10:g.83907050G>A	Likely pathogenic (PS4/PM2)	No	7.60 × 10^−08^	4/368	8%	1.35 × 10^−04^
Osteochondritis dissecans	2764	17:10505866_C/T (*MYH1*: missense) NC_000017.11:g.10505866C>T	Likely pathogenic (PS4/PM1)	No	1.01 × 10^−07^	3/56	3%	2.84 × 10^−04^
AA amyloidosis	85445	2:151727817_T/TGCTGGCTGTGCCAGA (*NEB:* disruptive inframe insertion) NC_000002.12:g.151727823_151727837dup	Likely pathogenic (PS4/PM4)	No	1.97 × 10^−07^	3/24	1%	7.56 × 10^−04^

The most significant associations involved the *JAK2* V617F variant, previously indicated as pathogenic for various diseases in ClinVar. We confirmed associations for polycythemia vera (*p = 1.32 × 10*^*−114*^), chronic myeloproliferative disease (*p = 2.89 × 10*^*−67*^), primary myelofibrosis (*p = 5.30 × 10*^*−40*^), and essential thrombocythemia (*p = 1.59 × 10*^*−12*^). Furthermore, we identified a significant V617F association for immune thrombocytopenic purpura (*p = 1.24 × 10*^*−13*^), which while not indicated in ClinVar or Orphanet, was previously reported in case studies from Poland [34] and Italy [35] and in mouse experiments [36]. Outside of *JAK2*, significant associations were identified involving three variants indicated as pathogenic or likely pathogenic by ClinVar: *HBB* with beta-thalassemia (*p = 7.34 × 10*^*−12*^), *F11* with congenital factor XI deficiency (*p = 3.41 × 10*^*−11*^), and *MYD88* with B-cell chronic lymphocytic leukemia (*p = 1.08 × 10*^*−9*^). The *HBB* gene encodes for β-globin, an important subunit of hemoglobin [37], while *F11* encodes for factor XI, which is needed for coagulation [38], and mutations in *MYD88* can induce oncogenesis through their impact on NFκB and JAK signaling regulation [39].

We also identified a significant association between *SRSF2* and chronic myelomonocytic leukemia (*p = 1.19 × 10*^*−13*^). Although *SRSF2* is a known gene for this disease (potentially causing oncogenesis through its impact on CD4/CD8 T-cells [40]) and was indicated by Orphanet, our variant (rs751713049) is novel. It is indicated as pathogenic (Additional file 1: Table S8) by four predictors (CADD, PROVEAN, MutationTester and SIFT), as well as possibly pathogenic by MutationAssessor and PolyPhen2 (using both HVAR and HDIV). A novel association was identified between *MYH1* and osteochondritis dissecans (*p = 1.01 × 10*^*−7*^), involving a variant predicted as pathogenic by seven predictors; *MYH1* is involved in skeletal muscle and has been linked to rhabdomyolysis [41].

We performed association analysis on the subset of variants predicted to cause loss of function in SnpEff (Additional file 2: Table S9). Nine significant associations were identified after Bonferroni adjustment, including a frameshift variant in *CALR* for essential thrombocythemia (*p = 1.59 × 10*^*−13*^) and the previously mentioned stop gained variant in *HBB* for beta-thalassemia. The *HBB* variant is recorded in ClinVar as pathogenic for beta-thalassemia, and the gene is annotated in Orphanet for this disease. While *CALR* is annotated in Orphanet as involved in essential thrombocythemia, our variant is novel.

## Discussion

Previous research has typically focused on specific rare conditions. Sun et al. [42] used ICD-9 codes to identify membranous nephropathy patients in the Kaiser Permanente health system, while Dickey et al. [43] used exome sequencing from the UKB to investigate whether erythropoietic protoporphyria may be under-diagnosed. The UKB [44] has also been analyzed more broadly, with rare variants in *JAK2* and *F11* associated to groups of myeloproliferative disease and congenital coagulation defects, respectively. Another study investigated extreme red blood cell indices in the UKB [45]. More recently, genetic association was performed for 33 rare diseases in data from 23andMe [46], and replication analysis confirmed significant associations for two diseases in the UKB.

Accurate disease identification is essential for expanding population-level biobank research to rare diseases. Although Orphanet indicated ICD-10 codes for > 6000 rare diseases, our consensus mapping approach revealed that only 566 of these were specific. ICD-10 codes are typically broader than their corresponding ORPHA codes and attempts to identify individuals using these codes would not be accurate for 92% of rare diseases. A previous study created a mapping from (the Australian modification of) ICD-10 codes to ORPHA codes [1] to investigate demographics in a public health system; however, it includes fewer rare diseases than our own; of the 14 significant variant-level associations (Table 2), only six involve a disease included in the Western Australia mapping. Other ontologies that have mappings for rare diseases include the UMLS [47], MonDO [10], and OMIM [48], and it is also possible to identify rare diseases by phenotype similarity [49]. We evaluated MonDO in our project by selecting the MonDO codes highest up the hierarchy (most general) for each ICD-10 code, then mapping to the exact match ORPHA code, where available (aiming for the most specific ORPHA code that is no more specific than the ICD-10 code). Of the 4682 ICD-10 codes mapped, 650 (14%) mapped to more than one ORPHA code, and some of the mappings did not meet our criteria. For example, A07.3 (isosporiasis) mapped to both ORPHA:472 (isosporiasis) and ORPHA:210 (cyclosporosis), which are caused by different parasites. A07.8 mapped to ORPHA:54368 (sarcocystosis), but its definition also includes other protozoal intestinal diseases. We therefore determined our consensus mapping approach was needed to identify specific rare diseases.

A single ICD-10 code can describe multiple diseases, so it is not always clear which disease an individual has, nor whether that disease is rare. Twenty-four of the diseases indicated as rare in Orphanet had greater than 1 in 2000 prevalence in the UKB; however, the UKB only recruited individuals aged 40–69, and this could lead to increased representation of rare diseases affecting that age range. To help ensure the diseases we identify are rare in Europe and the USA, we restricted our results to diseases reported by the NIH Genetic and Rare Diseases Information Center (GARD). Nevertheless, GARD leaves out some low prevalence diseases, such as cerebral sinovenous thrombosis (CSVT), while polymyalgia rheumatica is indicated as rare in both Orphanet and GARD but has a relatively high prevalence (~0.4%) in the UKB and Clinformatics®. Due to the challenges of diagnosing rare diseases, it is likely some individuals have a rare disease yet to be diagnosed.

Interestingly, many of the significant genetic associations we identified were for hematological diseases. While the proportion of hematological diseases we identified is large (Additional file 2: Figure S1), it is not the most frequent category; more neurological diseases were identified, yet we only found a significant genetic association for one of them (AA amyloidosis). Since the majority of UKB samples used for DNA extraction were taken from blood, some of the genetic associations we identified may be from de novo rather than hereditary variants. Identification of de novo variants is limited by the number of related individuals with rare diseases in the UKB. Of 24,416 individuals with a rare disease, 852 are estimated to be related to another individual in the UKB through genetics. Restricting to individuals with WES data, there are 22 pairs of related individuals where one has a rare disease. Of these, one pair includes an individual with Immune thrombocytopenic purpura and another includes an individual with B-cell chronic lymphocytic leukemia, however all individuals have the major allele for the associated variants.

Six of the significant (after Bonferroni correction) variant-level associations involve the *JAK2* V617F variant, for rare diseases that can be broadly categorized as myeloproliferative disorders. Among the 484 individuals who have at least one of these rare diseases, 72 of them (15%) have more than one of the six diseases, and it is plausible the mechanisms driving these associations may overlap [50]. Individuals with this variant had a slightly higher median age at recruitment (63 vs 58, Mann-Whitney *p* = 2.32 × 10^−12^), suggesting that the associated marker is a somatic mutation. Expanding our analysis to include variants with FDR< = 0.05 in genetic association (Additional file 1: Table S8), we found 40 significantly comorbid rare disease pairs that share an associated variant (Additional file 2: Table S10). In addition to the *JAK2* V617F variant pairs, immune thrombocytopenia, essential thrombocythemia, and chronic myeloproliferative disease were connected by *SELENON* G315S, which is indicated as pathogenic by ClinVar and has been associated with congenital myopathies among other diseases [51, 52].

## Conclusions

We have shown how consensus mapping between ICD and ORPHA codes can reveal relevant demographics and genetic associations for a wide range of rare diseases in different population-level datasets. By analyzing exome sequencing data from 167,246 individuals of European ancestry in the UKB, we confirmed and identified pathogenic variants for rare diseases, and as sample sizes continue to increase, the power available can lead to important discoveries. We have provided our findings, along with the ICD-10/ORPHA mapping in an interactive website to facilitate investigation of specific rare diseases.

Disease coding systems are constantly evolving, and ICD-11 now includes over 5000 rare diseases [53, 54]. When ICD-11 is widely implemented across clinics, future research will extend and repeat our consensus mapping approach, so that it can continue to enable rare disease research in population-level datasets. Our approach can therefore increase the sample size for rare conditions, especially those currently under-represented. The diverse range of information provided by the UKB (including health records, genetics, drug prescriptions, lifestyle, family/medical history etc.) can improve understanding of the overall burden of rare diseases.

## Supplementary Information

**Additional file 1: Table S1**: ICD-10 code to Orpha code consensus mapping. **Table S2**: Sample sizes and demographics obtained for rare disorders. **Table S4**: Significantly comorbid rare disease pairs (Bonferroni adjustment). **Table S5**: Complex diseases significantly comorbid with rare diseases (Bonferroni adjustment). **Table S7**: Significant gene-level associations (FDR<=0.05). **Table S8**: Significant variant-level associations (FDR<=0.05).

**Additional file 2: Figure S1**: Rare disease mapping and frequency of rare disease categories. **Figure S2**: Hexagon/scatter plot showing the mean age at recruitment and proportion of males for the diseases in each category. **Figure S3**: Sex of individuals with different groups of rare disease, grouped by age. **Figure S4**: Box/scatter plot of comorbidities for rare diseases, grouped by age. **Figure S5**: Heatmap showing the enrichment of comorbidities for individuals with specific groups of rare diseases compared to the full set of individuals with any rare disease. The colors represent odds ratios (OR) from Fisher exact tests, while asterisks indicate enrichments with significant p-values (after Bonferroni correction). **Figure S6**: Heatmap comparing the enrichment of comorbidities for individuals with specific groups of rare diseases with those from a previous study on comorbidities for individuals with common diseases^1^. The colors represent differences in odds ratios (OR) from Fisher exact tests. **Figure S7**: Heatmap showing the enrichment of complex disease comorbidities for individuals with 15 specific rare diseases included in the list of Mendelian diseases from a previous paper^2^. The colors represent log10 odds ratios (OR) from Fisher exact tests, while asterisks indicate enrichments with significant p-values (after Bonferroni correction). **Figure S8:** Histogram showing the number of ICD-10 codes mapping to different numbers of ORPHA codes in the original Orphanet mapping (in grey) as well as the number of these codes for which we were able to identify a single ORPHA code, such that individuals with the ICD-10 code should be expected to have the rare disease indicated by the ORPHA code. Some ICD-10 codes originally mapped to a large number of ORPHA codes, but across each of the bins, we were able to identify an appropriate single ORPHA code for a large proportion of ICD-10 codes, through our consensus mapping approach. **Table S3:** Comparing prevalence of in the UK Biobank and Optum. **Table S6**: Significant gene-level associations (Bonferroni adjustment). **Table S9:** Significant associations with loss of function variants (Bonferroni adjustment). **Table S10:** Shared variants between significantly comorbid rare diseases. **Supplementary Note:** Improvement in mapping through our consensus approach.

**Additional file 3.** Tutorial for using interactive website.

## Data Availability

Phenotypic data is provided through our interactive website http://www-personal.umich.edu/~mattpat/rareDiseases.html), and all information related to our mappings and significant genetic associations (adjusting for false discovery rate) are provided in supplementary material. Exome sequencing data of the UK Biobank is available to researchers on successful application and requires an access fee [55] (we downloaded the latest version available on 24 August 2020). The medical claims data used to demonstrate the transferability of our approach is available from the Clinformatics® Datamart available from Optum: optum.org/life-sciences-solutions (we used version 7.2). We obtained this data through the University of Michigan’s Institute for Healthcare Policy & Innovation (IHPI). The Clinformatics® Datamart includes a wide variety of data, including demographics, hospital admissions, procedures, and provider details; however, for the purposes of our study, we focused on the deidentified ICD-9/10 codes. Orphanet’s current mapping of ORPHA codes to ICD-10 codes is available online [56] (we downloaded the latest version available on 2 September 2020). The code from our analyses is available on GitHub [57].

## Abbreviations

GARD: NIH’s Genetic and Rare Diseases Information Center
ICD-10: 10th revision of the International Statistical Classification of Diseases and Related Health Problems
MAF: Minor allele frequency
UKB: UK Biobank
