# Supplementary material for: Enhanced rare disease mapping for phenome-wide genetic association in the UK Biobank
Source: Genome Med. 2022 Aug 9;14:85. doi: 10.1186/s13073-022-01094-y (PMC9364550; doi:10.1186/s13073-022-01094-y)
Supplement: Supplementary file 2 — Additional file 2: Figure S1: Rare disease mapping and frequency of rare disease categories. Figure S2: Hexagon/scatter plot showing the mean age at recruitment and proportion of males for the diseases in each category. Figure S3: Sex of individuals with different groups of rare disease, grouped by age. Figure S4: Box/scatter plot of comorbidities for rare diseases, grouped by age. Figure S5: Heatmap showing the enrichment of comorbidities for individuals with specific groups of rare diseases compared to the full set of individuals with any rare disease. The colors represent odds ratios (OR) from Fisher exact tests, while asterisks indicate enrichments with significant p-values (after Bonferroni correction). Figure S6: Heatmap comparing the enrichment of comorbidities for individuals with specific groups of rare diseases with those from a previous study on comorbidities for individuals with common diseases1. The colors represent differences in odds ratios (OR) from Fisher exact tests. Figure S7: Heatmap showing the enrichment of complex disease comorbidities for individuals with 15 specific rare diseases included in the list of Mendelian diseases from a previous paper2. The colors represent log10 odds ratios (OR) from Fisher exact tests, while asterisks indicate enrichments with significant p-values (after Bonferroni correction). Figure S8: Histogram showing the number of ICD-10 codes mapping to different numbers of ORPHA codes in the original Orphanet mapping (in grey) as well as the number of these codes for which we were able to identify a single ORPHA code, such that individuals with the ICD-10 code should be expected to have the rare disease indicated by the ORPHA code. Some ICD-10 codes originally mapped to a large number of ORPHA codes, but across each of the bins, we were able to identify an appropriate single ORPHA code for a large proportion of ICD-10 codes, through our consensus mapping approach. Table S3: Comparing prevalence of in the UK Biobank and Opt [file 13073_2022_1094_MOESM2_ESM.docx]

**Figure S1:** Rare disease mapping and frequency of rare disease categories.

a) Neoplasms (62 diseases) b) Musculoskeletal (34 diseases) c) Digestive system (12 diseases)

d) Congenital (106 diseases) e) Neurological (36 diseases) f) Blood (17 diseases)

g) Infectious/parasitic (51 diseases) h) Other Immune (6 diseases) i) Circulatory system (10 diseases)

j) Skin/Subcutaneous (33 diseases) k) Endocrine/metabolic (18 diseases) l) Genitourinary system (8 diseases)

m) Eye/ear (5 diseases) n) Pregnancy/childbirth (7 diseases) o) Respiratory system (5 diseases)

p) Miscellaneous (2 diseases) q) Injury/poisoning (8 diseases)

**Figure S2:** **Hexagon/scatter plot showing the mean age at recruitment and proportion of males for the diseases in each category.** The x-axis shows the percentage of males recorded as having each disease and the y-axis shows the mean age at recruitment. The hexagons show the density of diseases at each mean age and sex proportion, while the asterisks indicate the actual values for a particular disease. The red dashed line shows the overall mean age and sex proportion in the UK Biobank. Diseases for which there are no individuals in the UK Biobank with age or sex information cannot be plotted, so do not have asterisks.

a) Neoplasms (n=5,806) b) Musculoskeletal (n=4,446) c) Digestive system (n=3,397)

d) Congenital (n=2,162) e) Neurological (n=1,760) f) Blood (n=1,544)

g) Infectious/parasitic (n=939) h) Other Immune (n=933) i) Circulatory system (n=859)

j) Skin/Subcutaneous (n=721) k) Endocrine/metabolic (n=604) l) Genitourinary system (n=645)

m) Eye/ear (n=581) n) Pregnancy/childbirth (n=460) o) Respiratory system (n=306)

p) Miscellaneous (n=166) q) Injury/poisoning (n=91)

**Figure S3:** **Sex of individuals with different groups of rare disease, grouped by age.** Each bar plot presents the number of male and female individuals in the UK Biobank who have at least one rare disease from a particular group. Non-overlapping groups of rare diseases were identified from their corresponding ICD-10 chapters. Plots are grouped according to age at participation in the UK Biobank; the younger group (**red**) includes those under the mean age of 56, while the older group (**blue**) includes those above the mean age.

 **Figure S4: Box/scatter plot of comorbidities for rare diseases, grouped by age.** The y-axis shows, for each rare disease, the percentage of individuals who have at least one comorbidity in each group of diseases (excluding the rare disease itself). Plots are grouped according to age at participation in the UK Biobank; the younger group (**red**) includes those under the mean age of 56, while the older group (**blue**) includes those above the mean age.

**Figure S5:** Heatmap showing the enrichment of comorbidities for individuals with specific groups of rare diseases compared to the full set of individuals with any rare disease. The colors represent odds ratios (OR) from Fisher exact tests, while asterisks indicate enrichments with significant p-values (after Bonferroni correction).

**Figure S6:** Heatmap comparing the enrichment of comorbidities for individuals with specific groups of rare diseases with those from a previous study on comorbidities for individuals with common diseases^1^. The colors represent differences in odds ratios (OR) from Fisher exact tests.

**Figure S7:** Heatmap showing the enrichment of complex disease comorbidities for individuals with 15 specific rare diseases included in the list of Mendelian diseases from a previous paper^2^. The colors represent log10 odds ratios (OR) from Fisher exact tests, while asterisks indicate enrichments with significant p-values (after Bonferroni correction).

**Figure S8:** Histogram showing the number of ICD-10 codes mapping to different numbers of ORPHA codes in the original Orphanet mapping (in grey) as well as the number of these codes for which we were able to identify a single ORPHA code, such that individuals with the ICD-10 code should be expected to have the rare disease indicated by the ORPHA code. Some ICD-10 codes originally mapped to a large number of ORPHA codes, but across each of the bins, we were able to identify an appropriate single ORPHA code for a large proportion of ICD-10 codes, through our consensus mapping approach.

**Table S3:** Comparing prevalence of in the UK Biobank and Optum.

Abbreviations are as follows: Preval., prevalence. ^*^Diseases which were not listed on NIH’s GARD at the time of writing, so we were not able to confirm their rareness in the USA, although they are indicated as being rare in Europe by Orpha. ^†^Diseases which were listed as not being rare in the USA on NIH’s GARD.

|  |  | **UK Biobank** | | **Optum** **Clinformatics®** | |
| --- | --- | --- | --- | --- | --- |
| **ORPHA** | **Disease Name** | **Count** | **Preval. (%)** | **Count** | **Preval. (%)** |
| 131 | Budd-Chiari syndrome *(ICD-10: I82.0, ICD-9: 453.0)* | 22 | 4.38E-03 | 8277 | 2.14E-02 |
| 150 | Nasopharyngeal carcinoma *(ICD-10: C11.0, ICD-9: 733.7)* | 16 | 3.18E-03 | 5452 | 1.41E-02 |
| 173 | Cholera *(ICD-10: A00.[0,1,9], ICD-9: 001.[0,1,9])* | 4 | 7.96E-04 | 8057 | 2.08E-02 |
| 223 | Nephrogenic diabetes insipidus *(ICD-10: N25.1, ICD-9: 588.1)* | 20 | 3.98E-03 | 7697 | 1.99E-02 |
| 288 | Hereditary elliptocytosis *(ICD-10: D58.1, ICD-9: 282.1)* | 8 | 1.59E-03 | 2488 | 6.42E-03 |
| 290 | Congenital rubella syndrome *(ICD-10: P35.0, ICD-9: 771.0)* | 2 | 3.98E-04 | 578 | 1.49E-03 |
| 318 | Acute erythroid leukemia *(ICD-10: C94.0[0,1,2], ICD-9: 207.0[0,1,2])* | 3 | 5.97E-04 | 1616 | 4.17E-03 |
| 329 | Congenital factor XI deficiency *(ICD-10: D68.1, ICD-9: 286.2)* | 34 | 6.77E-03 | 5547 | 1.43E-02 |
| 399 | Huntington disease *(ICD-10: G10, ICD-9: 333.4)* | 28 | 5.57E-03 | 3859 | 9.96E-03 |
| 422 | Idiopathic/heritable pulmonary arterial hypertension *(ICD-10: I27.0, ICD-9: 416.0)* | 640 | 1.27E-01 | 212802 | 5.49E-01 |
| 549 | Legionellosis *(ICD-10: A48.1, ICD-9: 482.84)* | 77 | 1.53E-02 | 3997 | 1.03E-02 |
| 567 | 22q11.2 deletion syndrome *(ICD-10: D82.1, ICD-9: 279.11)* | 18 | 3.58E-03 | 2045 | 5.28E-03 |
| 589 | Myasthenia gravis *(ICD-10: G70.0[0,1], ICD-9: 358.0[0,1])* | 266 | 5.29E-02 | 35291 | 9.11E-02 |
| 660 | Omphalocele* *(ICD-10: Q79.2, ICD-9: 756.72)* | 1 | 1.99E-04 | 1267 | 3.27E-03 |
| 666 | Osteogenesis imperfecta *(ICD-10: Q78.0, ICD-9: 756.51)* | 48 | 9.55E-03 | 4515 | 1.17E-02 |
| 730 | Autosomal dominant polycystic kidney disease^†^ *(ICD-10: Q61.2, ICD-9: 753.13)* | 208 | 4.14E-02 | 14124 | 3.65E-02 |
| 774 | Hereditary hemorrhagic telangiectasia *(ICD-10: I78.0, ICD-9: 448.0)* | 117 | 2.33E-02 | 13853 | 3.58E-02 |
| 781 | Q fever *(ICD-10: A78, ICD-9: 083.0)* | 2 | 3.98E-04 | 2794 | 7.21E-03 |
| 805 | Tuberous sclerosis complex *(ICD-10: Q85.1, ICD-9: 759.5)* | 12 | 2.39E-03 | 4764 | 1.23E-02 |
| 810 | Shigellosis *(ICD-10: A03.[0,1,2,3,8,9], ICD-9: 004.[0,1,2,3,8,9])* | 18 | 3.58E-03 | 6637 | 1.71E-02 |
| 822 | Hereditary spherocytosis *(ICD-10: D58.0, ICD-9: 282.0)* | 54 | 1.07E-02 | 9502 | 2.45E-02 |
| 846 | Alpha-thalassemia *(ICD-10: D56.0, ICD-9: 282.43)* | 33 | 6.57E-03 | 2965 | 7.65E-03 |
| 848 | Beta-thalassemia *(ICD-10: D56.1, ICD-9: 282.44)* | 71 | 1.41E-02 | 4041 | 1.04E-02 |
| 903 | Von Willebrand disease^†^ *(ICD-10: D68.0, ICD-9: 286.4)* | 152 | 3.02E-02 | 19559 | 5.05E-02 |
| 908 | Fragile X syndrome *(ICD-10: Q99.2, ICD-9: 759.83)* | 3 | 5.97E-04 | 5524 | 1.43E-02 |
| 1077 | Dental ankylosis *(ICD-10: K03.5, ICD-9: 521.6)* | 5 | 9.95E-04 | 921 | 2.38E-03 |
| 1247 | Schistosomiasis *(ICD-10: B65.[0,1,2,3,8,9], ICD-9: 120.[0,1,2,3,8,9])* | 13 | 2.59E-03 | 4959 | 1.28E-02 |
| 1304 | Brucellosis *(ICD-10: A23.[0,1,2,3,8,9], ICD-9: 023.[0,1,2,3,8,9])* | 1 | 1.99E-04 | 1101 | 2.84E-03 |
| 1457 | Aorta coarctation *(ICD-10: Q25.1, ICD-9: 747.10)* | 35 | 6.97E-03 | 11961 | 3.09E-02 |
| 1464 | Univentricular heart* *(ICD-10: Q20.4, ICD-9: 745.3)* | 2 | 3.98E-04 | 2761 | 7.13E-03 |
| 1478 | Interatrial communication* *(ICD-10: Q21.2, ICD-9: 745.5)* | 654 | 1.30E-01 | 168537 | 4.35E-01 |
| 1549 | Cryptosporidiosis *(ICD-10: A07.2, ICD-9: 007.4)* | 12 | 2.39E-03 | 2552 | 6.59E-03 |
| 1656 | Dermatitis herpetiformis *(ICD-10: L13.0, ICD-9: 694.0)* | 46 | 9.15E-03 | 14418 | 3.72E-02 |
| 1880 | Ebstein malformation *(ICD-10: Q22.5, ICD-9: 746.2)* | 18 | 3.58E-03 | 3241 | 8.37E-03 |
| 2022 | Endocardial fibroelastosis *(ICD-10: I42.4, ICD-9: 425.3)* | 13 | 2.59E-03 | 3228 | 8.33E-03 |
| 2073 | Narcolepsy type 1 *(ICD-10: G474.[11,19,21,29], ICD-9: 347.[11,19,21,29])* | 70 | 1.39E-02 | 58208 | 1.50E-01 |
| 2137 | Autoimmune hepatitis *(ICD-10: K75.4, ICD-9: 571.42)* | 236 | 4.70E-02 | 15647 | 4.04E-02 |
| 2248 | Hypoplastic left heart syndrome *(ICD-10: Q23.4, ICD-9: 746.7)* | 2 | 3.98E-04 | 3217 | 8.31E-03 |
| 2331 | Kawasaki disease *(ICD-10: M30.3, ICD-9: 446.1)* | 3 | 5.97E-04 | 10227 | 2.64E-02 |
| 2374 | Congenital laryngeal web* *(ICD-10: Q31.0, ICD-9: 748.2)* | 14 | 2.79E-03 | 1158 | 2.99E-03 |
| 2444 | Congenital pulmonary airway malformation* *(ICD-10: Q33.0, ICD-9: 748.4)* | 1 | 1.99E-04 | 1670 | 4.31E-03 |
| 2573 | Moyamoya disease *(ICD-10: I67.5, ICD-9: 437.5)* | 3 | 5.97E-04 | 1864 | 4.81E-03 |
| 3092 | Fixed subaortic stenosis *(ICD-10: Q24.4, ICD-9: 746.81)* | 8 | 1.59E-03 | 4805 | 1.24E-02 |
| 3093 | Congenital aortic valve stenosis *(ICD-10: Q23.0, ICD-9: 746.3)* | 46 | 9.15E-03 | 16853 | 4.35E-02 |
| 3096 | Reye syndrome *(ICD-10: G93.7, ICD-9: 331.81)* | 1 | 1.99E-04 | 653 | 1.69E-03 |
| 3099 | Rheumatic fever (ICD-10: I0[0,1.0,1.1,1.2,1.8,1.9], ICD-9: 39[0,1.0,1.1,1.2,1.8,1.9]) | 37 | 7.36E-03 | 22054 | 5.69E-02 |
| 3189 | Congenital pulmonary valve stenosis *(ICD-10: Q22.1, ICD-9: 746.02)* | 16 | 3.18E-03 | 16286 | 4.20E-02 |
| 3190 | Subpulmonary stenosis *(ICD-10: Q24.3, ICD-9: 746.83)* | 4 | 7.96E-04 | 2179 | 5.63E-03 |
| 3287 | Takayasu arteritis *(ICD-10: M31.4, ICD-9: 44.67)* | 11 | 2.19E-03 | 3067 | 7.92E-03 |
| 3303 | Tetralogy of Fallot *(ICD-10: Q21.3, ICD-9: 745.2)* | 19 | 3.78E-03 | 8699 | 2.25E-02 |
| 3384 | Truncus arteriosus *(ICD-10: 33.84, ICD-9: 745.0)* | 2 | 3.98E-04 | 3512 | 9.07E-03 |
| 3426 | Double outlet right ventricle *(ICD-10: Q20.1, ICD-9: 745.11)* | 3 | 5.97E-04 | 2561 | 6.61E-03 |
| 29073 | Multiple myeloma *(ICD-10: C90.0[0,1,2], ICD-9: 203.0[0,1,2])* | 855 | 1.70E-01 | 66490 | 1.72E-01 |
| 33276 | Kaposi sarcoma *(ICD-10: C46.[0,1,2,3,5,7,8,9], ICD-9: 176.[0,1,2,3,5,7,8,9])* | 13 | 2.59E-03 | 6655 | 1.72E-02 |
| 36234 | Bacterial toxic-shock syndrome *(ICD-10: A48.3, ICD-9: 040.82)* | 10 | 1.99E-03 | 1974 | 5.10E-03 |
| 36238 | Staphylococcal necrotizing pneumonia* *(ICD-10: J15.2[0,1,2,9], ICD-9:482.4[0,1,2,9])* | 111 | 2.21E-02 | 52437 | 1.35E-01 |
| 36258 | Buerger disease *(ICD-10: I73.1, ICD-9: 443.1)* | 17 | 3.38E-03 | 9025 | 2.33E-02 |
| 36426 | Stevens-Johnson syndrome *(ICD-10: L51.1, ICD-9: 695.13)* | 51 | 1.01E-02 | 5023 | 1.30E-02 |
| 43393 | Lambert-Eaton myasthenic syndrome *(ICD-10: G73.1, ICD-9: 358.31)* | 4 | 7.96E-04 | 173 | 4.47E-04 |
| 48104 | Pyoderma gangrenosum *(ICD-10: L88, ICD-9: 686.01)* | 40 | 7.96E-03 | 8644 | 2.23E-02 |
| 48377 | Subcorneal pustular dermatosis *(ICD-10: L13.1, ICD-9: 694.1)* | 5 | 9.95E-04 | 1411 | 3.64E-03 |
| 50839 | Cat-scratch disease *(ICD-10: A28.1, ICD-9: 078.3)* | 5 | 9.95E-04 | 11377 | 2.94E-02 |
| 55655 | Pneumococcal meningitis *(ICD-10: G00.1, ICD-9: 320.1)* | 63 | 1.25E-02 | 1647 | 4.25E-03 |
| 60041 | Congenital heart block *(ICD-10: Q24.6, ICD-9: 746.86)* | 13 | 2.59E-03 | 2831 | 7.31E-03 |
| 67038 | B-cell chronic lymphocytic leukemia *(ICD-10: C91.1[0,1,2], ICD-9: 204.1[0,1,2])* | 752 | 1.50E-01 | 61119 | 1.58E-01 |
| 70567 | Cholangiocarcinoma *(ICD-10: C22.1, ICD-9: 155.1)* | 274 | 5.45E-02 | 11965 | 3.09E-02 |
| 71211 | Neuromyelitis optica *(ICD-10: G36.0, ICD-9: 341.0)* | 20 | 3.98E-03 | 3711 | 9.58E-03 |
| 83314 | Epidemic typhus *(ICD-10: A75.0, ICD-9: 080)* | 1 | 1.99E-04 | 378 | 9.76E-04 |
| 83317 | Scrub typhus *(ICD-10: A75.3, ICD-9: 081.2)* | 1 | 1.99E-04 | 122 | 3.15E-04 |
| 83330 | Proximal spinal muscular atrophy type 1 *(ICD-10: G12.0, ICD-9: 335.0)* | 1 | 1.99E-04 | 1173 | 3.03E-03 |
| 83461 | Congenital primary aphakia *(ICD-10: Q12.3, ICD-9: 743.35)* | 2 | 3.98E-04 | 430 | 1.11E-03 |
| 83463 | Microtia* *(ICD-10: Q17.2, ICD-9: 744.23)* | 4 | 7.96E-04 | 2583 | 6.67E-03 |
| 86850 | Myeloid sarcoma *(ICD-10: C92.3[0,1,2], ICD-9: 205.3[0,1,2])* | 4 | 7.96E-04 | 1291 | 3.33E-03 |
| 90042 | Primary familial polycythemia *(ICD-10: D75.0, ICD-9: 289.6)* | 37 | 7.36E-03 | 13694 | 3.54E-02 |
| 90066 | Pneumonia caused by Pseudomonas aeruginosa infection *(ICD-10: J15.1, 482.1)* | 144 | 2.87E-02 | 30461 | 7.86E-02 |
| 91411 | Congenital ptosis* *(ICD-10: J15.1, ICD-9: 482.1)* | 31 | 6.17E-03 | 14998 | 3.87E-02 |
| 91495 | Persistent hyperplastic primary vitreous* *(ICD-10: Q14.0, ICD-9: 743.51)* | 2 | 3.98E-04 | 1528 | 3.94E-03 |
| 91547 | Relapsing fever* *(ICD-10: A68.[0,1,9], ICD-9: 087.[0,1,9])* | 1 | 1.99E-04 | 10133 | 2.62E-02 |
| 93108 | Renal dysplasia* *(ICD-10: Q61.4, ICD-9: 753.15)* | 16 | 3.18E-03 | 4420 | 1.14E-02 |
| 93276 | Polyostotic fibrous dysplasia *(ICD-10: Q78.1, ICD-9: 756.54)* | 5 | 9.95E-04 | 1186 | 3.06E-03 |
| 93569 | Polymyalgia rheumatica *(ICD-10: M35.3, ICD-9: 725)* | 1961 | 3.90E-01 | 147287 | 3.80E-01 |
| 93958 | Oromandibular dystonia* *(ICD-10: G24.4, ICD-9: 333.82)* | 10 | 1.99E-03 | 9407 | 2.43E-02 |
| 94093 | Neuroleptic malignant syndrome *(ICD-10: G21.0, ICD-9: 333.92)* | 11 | 2.19E-03 | 1543 | 3.98E-03 |
| 96269 | Isolated partial vaginal agenesis* *(ICD-10: Q52.0, ICD-9: 752.45)* | 3 | 5.97E-04 | 454 | 1.17E-03 |
| 97292 | Cardiogenic shock* *(ICD-10: R57.0, ICD-9: 785.51)* | 163 | 3.24E-02 | 76203 | 1.97E-01 |
| 97352 | Pellagra *(ICD-10: E52, ICD-9: 265.2)* | 3 | 5.97E-04 | 1383 | 3.57E-03 |
| 98823 | Chronic myelomonocytic leukemia *(ICD-10: C93.1[0,1,2], ICD-9: 206.1[0,1,2])* | 51 | 1.01E-02 | 4283 | 1.11E-02 |
| 98878 | Hemophilia A *(ICD-10: D66, ICD-9: 286.0)* | 94 | 1.87E-02 | 16151 | 4.17E-02 |
| 98879 | Hemophilia B *(ICD-10: D67, ICD-9: 286.1)* | 21 | 4.18E-03 | 3415 | 8.82E-03 |
| 99057 | Congenital mitral stenosis *(ICD-10: Q23.2, ICD-9: 746.5)* | 1 | 1.99E-04 | 3310 | 8.55E-03 |
| 99797 | Anodontia *(ICD-10: K00.0, ICD-9: 520.0)* | 33 | 6.57E-03 | 6699 | 1.73E-02 |
| 99906 | Farmer's lung disease *(ICD-10: J67.0, ICD-9: 495.0)* | 7 | 1.39E-03 | 1654 | 4.27E-03 |
| 99908 | Pigeon-breeder lung disease *(ICD-10: J67.2, ICD-9: 495.2)* | 24 | 4.78E-03 | 893 | 2.31E-03 |
| 100093 | Carcinoid syndrome *(ICD-10: E34.0, ICD-9: 259.2)* | 59 | 1.17E-02 | 12999 | 3.36E-02 |
| 137583 | Vulvar intraepithelial neoplasia* *(ICD-10: D07.1, ICD-9: 233.32)* | 149 | 2.97E-02 | 11145 | 2.88E-02 |
| 137686 | Asherman syndrome *(ICD-10: N85.6, ICD-9: 621.5)* | 110 | 2.19E-02 | 23979 | 6.19E-02 |
| 137820 | Extrapelvic endometriosis* *(ICD-10: N80.[5,6], ICD-9: 617.[5,6])* | 77 | 1.53E-02 | 11092 | 2.86E-02 |
| 137914 | Choanal atresia* *(ICD-19: Q30.0, ICD-9: 748.0)* | 5 | 9.95E-04 | 3615 | 9.33E-03 |
| 183663 | Hyper-IgM syndrome with susceptibility to opportunistic infections *(ICD-10: D80.5, ICD-9: 279.05)* | 3 | 5.97E-04 | 1290 | 3.33E-03 |
| 221091 | Trigeminal neuralgia *(ICD-10: G50.0, ICD-9: 350.1)* | 586 | 1.17E-01 | 103382 | 2.67E-01 |
| 231237 | Delta-beta-thalassemia *(ICD-10: D56.2, ICD-9: 282.45)* | 3 | 5.97E-04 | 140 | 3.61E-04 |
| 238468 | Hypohidrotic ectodermal dysplasia *(ICD-10: Q82.4, ICD-9: 747.31)* | 1 | 1.99E-04 | 2456 | 6.34E-03 |
| 238624 | Idiopathic intracranial hypertension *(ICD-10: G93.2, ICD-9: 348.2)* | 132 | 2.63E-02 | 33396 | 8.62E-02 |
| 276145 | Malignant epithelial tumor of salivary glands *(ICD-10: C0[7,8.0,8.1], ICD-9: 142.[0,1,2])* | 134 | 2.67E-02 | 14709 | 3.80E-02 |
| 289157 | Hypocalcemic vitamin D-dependent rickets *(ICD-10: E55.0, ICD-9: 268.0)* | 6 | 1.19E-03 | 29654 | 7.66E-02 |
| 293208 | Celiac artery compression syndrome *(ICD-10: I77.4, ICD-9: 447.4)* | 5 | 9.95E-04 | 17090 | 4.41E-02 |
| 306731 | Sydenham chorea *(ICD-10: I02.[0,9], ICD-9: 392.[0,9])* | 2 | 3.98E-04 | 5772 | 1.49E-02 |
| 329217 | Cerebral sinovenous thrombosis* *(ICD-10: I67.6, ICD-9: 437.6)* | 7 | 1.39E-03 | 2713 | 7.00E-03 |
| 331235 | Selective IgM deficiency *(ICD-10: D80.4, ICD-9: 279.02)* | 18 | 3.58E-03 | 3083 | 7.96E-03 |
| 353253 | Burning mouth syndrome^†^ *(ICD-10: K14.6, ICD-9: 529.6)* | 38 | 7.56E-03 | 45340 | 1.17E-01 |
| 357154 | Oral submucous fibrosis *(ICD-10: K13.5, ICD-9: 528.8)* | 23 | 4.58E-03 | 6797 | 1.75E-02 |
| 411703 | Pulmonary non-tuberculous mycobacterial infection *(ICD-10: A31.0, ICD-9: 031.0)* | 41 | 8.16E-03 | 17819 | 4.60E-02 |
| 443227 | Paratyphoid fever *(ICD-10: A01.[1,2,3,4], ICD-9: 002.[1,2,3,9])* | 8 | 1.59E-03 | 1031 | 2.66E-03 |
| 454714 | Plasma cell leukemia *(ICD-10: C90.1[0,1,2], ICD-9: 203.1[0,1,2])* | 26 | 5.17E-03 | 3151 | 8.13E-03 |

**Table S6:** Significant gene-level associations (Bonferroni adjustment).
Gene associations are indicated as being ‘Known’ if the gene is annotated for that disease in Orpha, OMIM, MedGen, Mondo, ClinVar or ClinGen, otherwise they are indicated as ‘Novel’. The p-values provided are from SAIGE-GENE’s SKAT-O test, with #Markers specifying the number of markers included in each gene-based test. Although a significant gene-level association was identified for interstitial cystitis, this disease was indicated as not being rare in the USA by NIH’s GARD. Furthermore, significant gene-level associations with preeclampsia, non-syndromic genetic deafness, and interatrial communication were excluded because these diseases were not listed on NIH’s GARD, so it is difficult to confirm their rareness in the USA. See **Supplementary Table 4** for the full set of gene-level associations (FDR<=0.05) with diseases indicated as being rare in Europe by Orpha. **MUC4* associations can potentially be affected by multiple mapping reads, and using k=50 mappability estimates, we identified a small number of markers for each association in non-unique regions (Immune thrombocytopenic purpura: 9/35; Chronic myeloproliferative disease: 2/14; Polycythemia vera: 11/39; B-cell chronic lymphocytic leukemia: 27/85; Follicular lymphoma: 17/51; Trigeminal neuralgia: 14/54).

| **Disease** | **Orpha** | **Gene** | **Known?** | **P-Value** | **#Markers** |
| --- | --- | --- | --- | --- | --- |
| Essential thrombocythemia | 3318 | *JAK2* | Known | 1.78E-32 | 2 |
| Immune thrombocytopenic purpura | 3002 | *JAK2* | Novel | 1.24E-13 | 2 |
| Essential thrombocythemia | 3318 | *TRIOBP* | Novel | 1.10E-09 | 2 |
| Giant cell arteritis | 397 | *CCDC88A* | Novel | 6.32E-09 | 2 |
| Systemic sclerosis | 90291 | *FCGBP* | Novel | 6.65E-09 | 3 |
| Immune thrombocytopenic purpura | 3002 | *MUC4^*^* | Novel | 7.18E-08 | 35 |
| Chronic myeloproliferative disease | 86830 | *MUC4^*^* | Novel | 4.07E-07 | 14 |
| Aspergillosis | 1163 | *TTN* | Novel | 8.67E-07 | 11 |
| Polycythemia vera | 729 | *MUC4^*^* | Novel | 9.08E-07 | 39 |
| Autoimmune hepatitis | 2137 | *KCNK16* | Novel | 1.22E-06 | 3 |
| B-cell chronic lymphocytic leukemia | 67038 | *MUC4^*^* | Novel | 1.23E-06 | 85 |
| Chronic myeloid leukemia | 521 | *CDH24* | Novel | 1.29E-06 | 2 |
| Follicular lymphoma | 545 | *MUC4^*^* | Novel | 1.67E-06 | 51 |
| Trigeminal neuralgia | 221091 | *MUC4^*^* | Novel | 1.69E-06 | 54 |

**Table S9:** Significant associations with loss of function variants (Bonferroni adjustment).
Markers are indicated using their chromosome and position (in hg38 build), then the major (non-risk) allele and minor (risk) allele. Associations were conducted at the variant-level using SAIGE. Associations are indicated as being ‘Known’ if the gene that has loss of function is annotated for that disease in Orpha, otherwise they are indicated as ‘Novel’. *Our association is for a different variant compared to the one indicated for that disease/gene in ClinVar. ^†^Diseases which were not listed on NIH’s GARD at the time of writing, so we were not able to confirm their rareness in the USA, although they are indicated as being rare in Europe by Orpha.

Abbreviations are as follows: E., Essential; W., Waldenström; N., Nephrogenic; M.T., Malignant tumor; MAC, minor allele count; MAF, minor allele frequency; LOF, percentage loss of function predicted by SnpEff.

| **Disease** | **Orpha** | **Marker** | **Case MAC** | **Control MAF** | **P-value** | **Gene** | **Known?** | **LOF** |
| --- | --- | --- | --- | --- | --- | --- | --- | --- |
| E. thrombocythemia | 3318 | 19:12943813_A/ATTGTC (frameshift) | 5/218 | 1.50E-05 | 2.82E-13 | *CALR* | Known* | 14% |
| Beta-thalassemia | 848 | 11:5226774_G/A (stop gained) | 3/12 | 1.79E-05 | 3.46E-12 | *HBB* | Known | 33% |
| Polycythemia vera | 729 | 9:122568173_ATC/A (frameshift) | 3/370 | 7.78E-05 | 6.69E-07 | *OR1L8* | Novel | 100% |
| Endophthalmitis^†^ | 199323 | 15:45100230_T/C (splice acceptor) | 3/118 | 2.36E-04 | 9.84E-07 | *DUOX1* | Novel | 40% |
| W. macroglobulinemia | 33226 | 9:99020443_G/T (splice donor) | 3/84 | 4.93E-04 | 3.56E-06 | *COL15A1* | Novel | 40% |
| Solar urticaria^†^ | 97230 | 19:6381617_T/C (splice acceptor) | 3/8 | 6.16E-03 | 5.42E-06 | *GTF2F1* | Novel | 50% |
| N. diabetes insipidus | 223 | 3:108362328_C/CT (splice acceptor) | 3/18 | 3.08E-03 | 5.80E-06 | *HHLA2* | Novel | 64% |
| Multiple myeloma | 29073 | 12:53329305_TTC/T (frameshift) | 3/532 | 8.68E-05 | 7.03E-06 | *SP7* | Novel | 100% |
| M.T. of fallopian tubes | 180242 | 2:219329210_C/A (stop gained) | 3/50 | 1.18E-03 | 7.97E-06 | *RESP18* | Novel | 33% |

**Table S10:** Shared variants between significantly comorbid rare diseases.

| **Variant** | **Rare Disease 1** | **Orpha 1** | **Rare Disease 2** | **Orpha 2** |
| --- | --- | --- | --- | --- |
| 1:25809753_G/A | Essential thrombocythemia | 3318 | Chronic myeloproliferative disease | 86830 |
| 1:25809753_G/A | Immune thrombocytopenic purpura | 3002 | Chronic myeloproliferative disease | 86830 |
| 1:25809753_G/A | Immune thrombocytopenic purpura | 3002 | Essential thrombocythemia | 3318 |
| 1:152761247_G/GC | Immune thrombocytopenic purpura | 3002 | Essential thrombocythemia | 3318 |
| 1:226383066_G/A | Primary biliary cholangitis | 186 | Systemic sclerosis | 90291 |
| 1:226383066_G/A | Systemic lupus erythematosus | 536 | Systemic sclerosis | 90291 |
| 2:85665785_C/T | Polycythemia vera | 729 | Chronic myeloproliferative disease | 86830 |
| 2:214987674_T/C | Polycythemia vera | 729 | Immune thrombocytopenic purpura | 3002 |
| 3:123291187_C/T | Polycythemia vera | 729 | Essential thrombocythemia | 3318 |
| 4:2445228_A/T | Renal agenesis, unilateral | 93100 | Renal agenesis | 411709 |
| 5:10250426_T/C | Systemic lupus erythematosus | 536 | Discoid lupus erythematosus | 90281 |
| 6:28363334_A/ATGC | Polycythemia vera | 729 | Immune thrombocytopenic purpura | 3002 |
| 6:32522142_G/A | Essential thrombocythemia | 3318 | Chronic myeloproliferative disease | 86830 |
| 7:5960821_G/A | Polycythemia vera | 729 | Chronic myeloproliferative disease | 86830 |
| 8:141218043_T/C | Systemic lupus erythematosus | 536 | Discoid lupus erythematosus | 90281 |
| 8:143538158_A/C | Polycythemia vera | 729 | Essential thrombocythemia | 3318 |
| 9:5073770_G/T | Chronic myeloproliferative disease | 86830 | Acute panmyelosis with myelofibrosis | 86843 |
| 9:5073770_G/T | Essential thrombocythemia | 3318 | Acute panmyelosis with myelofibrosis | 86843 |
| 9:5073770_G/T | Essential thrombocythemia | 3318 | Chronic myeloproliferative disease | 86830 |
| 9:5073770_G/T | Immune thrombocytopenic purpura | 3002 | Chronic myeloproliferative disease | 86830 |
| 9:5073770_G/T | Immune thrombocytopenic purpura | 3002 | Essential thrombocythemia | 3318 |
| 9:5073770_G/T | Polycythemia vera | 729 | Chronic myeloproliferative disease | 86830 |
| 9:5073770_G/T | Polycythemia vera | 729 | Essential thrombocythemia | 3318 |
| 9:5073770_G/T | Polycythemia vera | 729 | Immune thrombocytopenic purpura | 3002 |
| 9:5073770_G/T | Polycythemia vera | 729 | Primary myelofibrosis | 824 |
| 9:5073770_G/T | Primary myelofibrosis | 824 | Acute panmyelosis with myelofibrosis | 86843 |
| 9:5073770_G/T | Primary myelofibrosis | 824 | Chronic myeloproliferative disease | 86830 |
| 9:5073770_G/T | Primary myelofibrosis | 824 | Essential thrombocythemia | 3318 |
| 9:5073770_G/T | Primary myelofibrosis | 824 | Immune thrombocytopenic purpura | 3002 |
| 9:96301972_G/A | Primary biliary cholangitis | 186 | Autoimmune hepatitis | 2137 |
| 9:114331843_A/T | Pulmonary arterial hypertension | 422 | Sarcoidosis | 797 |
| 11:315073_A/G | Chronic myeloid leukemia | 521 | B-cell chronic lymphocytic leukemia | 67038 |
| 11:46680520_G/A | CREST syndrome | 90290 | Systemic sclerosis | 90291 |
| 12:6938748_G/A | Chronic myeloid leukemia | 521 | B-cell chronic lymphocytic leukemia | 67038 |
| 12:55693284_C/T | Giant cell arteritis | 397 | Polymyalgia rheumatica | 93569 |
| 16:31038016_C/T | Immune thrombocytopenic purpura | 3002 | Essential thrombocythemia | 3318 |
| 19:549063_C/T | Systemic lupus erythematosus | 536 | Discoid lupus erythematosus | 90281 |
| 19:758613_G/A | Immune thrombocytopenic purpura | 3002 | Chronic myeloproliferative disease | 86830 |
| 19:10554197_G/C | Systemic lupus erythematosus | 536 | Systemic sclerosis | 90291 |
| 21:41494370_G/A | Pulmonary arterial hypertension | 422 | CREST syndrome | 90290 |

**Supplementary Note:** Improvement in mapping through our consensus approach

Our mapping provides a more precise and less ambiguous description of each rare disease (**Supplementary Figure 3**). For 982 out of 2,044 ICD-10 codes originally mapping to more than one ORPHA code, we are able to refine the rare disease mapping for 526 ICD-10 codes, allowing them to have specific definition for 391 ORPHA codes. Interestingly, we showed 456 of the 982 ICD10 codes had no ORPHA codes that mapped exactly to the disease, and we do not include these in our subsequent analyses. For example, we mapped ICD-10 D56.1, annotated for 71 individuals in the UKB, to ORPHA:848 (beta-thalassemia). Alternatively, Orphanet maps ORPHA:68364 (hemoglobinopathy) and ORPHA:275749 (beta-thalassemia and related diseases) to D56.1, however these groups of disorders are less specific than ORPHA:848; similarly ORPHA:231214 (beta-thalassemia major), ORPHA:231222 (beta-thalassemia intermedia) and ORPHA:231226 (dominant beta-thalassemia) are also mapped to D56.1, however these are disease subtypes. We therefore selected ORPHA:848 as the code to map from D56.1. Of 6,066 rare diseases suggested by the original Orphnet mapping, 756 (12%) have greater than one in 2,000 prevalence (the European criterion for rarity) in the UK Biobank. By comparison, our refined mapping identified patients with 420 rare diseases, of which 24 (6%) have greater than one in 2,000 prevalence, demonstrating that our approach can help to improve the accuracy of identifying rare diseases.

**References**

1 Dong, G., Feng, J., Sun, F., Chen, J. & Zhao, X. M. A global overview of genetically interpretable multimorbidities among common diseases in the UK Biobank. *Genome Med* **13**, 110, doi:10.1186/s13073-021-00927-6 (2021).

2 Blair, D. R. *et al.* A nondegenerate code of deleterious variants in Mendelian loci contributes to complex disease risk. *Cell* **155**, 70-80, doi:10.1016/j.cell.2013.08.030 (2013).
