# Supplementary material for: Enhanced rare disease mapping for phenome-wide genetic association in the UK Biobank
Source: Genome Med. 2022 Aug 9;14:85. doi: 10.1186/s13073-022-01094-y (PMC9364550; doi:10.1186/s13073-022-01094-y)
Supplement: Supplementary file 3 — Additional file 3. Tutorial for using interactive website. [file 13073_2022_1094_MOESM3_ESM.docx]

**Tutorial for using interactive website for rare diseases in the UK Biobank**

1. Select a disease from the rare disease table. The table consists of four columns: Disease Name (from OrphaNet), ORPHA Number (from OrphaNet), ICD10 Codes (from our consensus ICD-10 to ORPHA number mapping), UKB Count (the number of individuals in the UK Biobank indicated as having the rare disease by our mapping) and Group (determined from the ICD-10 chapters).


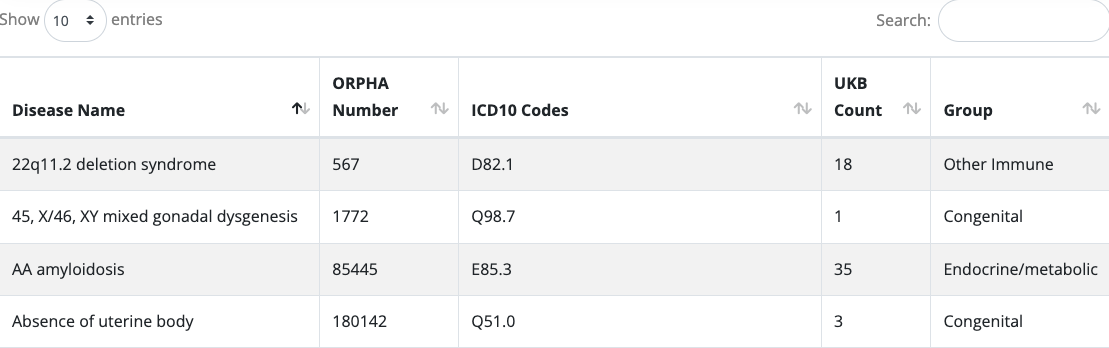


1. You can change the number of entries shown in a single screen by selecting a different value from the “Show XX entries” drop-down box. It is also possible to search for specific rare diseases using the “Search” box or sort by the values in each column by clicking on the up/down arrows next to the column header. At the bottom of the table, is a navigation tool, which can be used to move to subsequent rare diseases, either by clicking on the screen number, or the “Next” button.


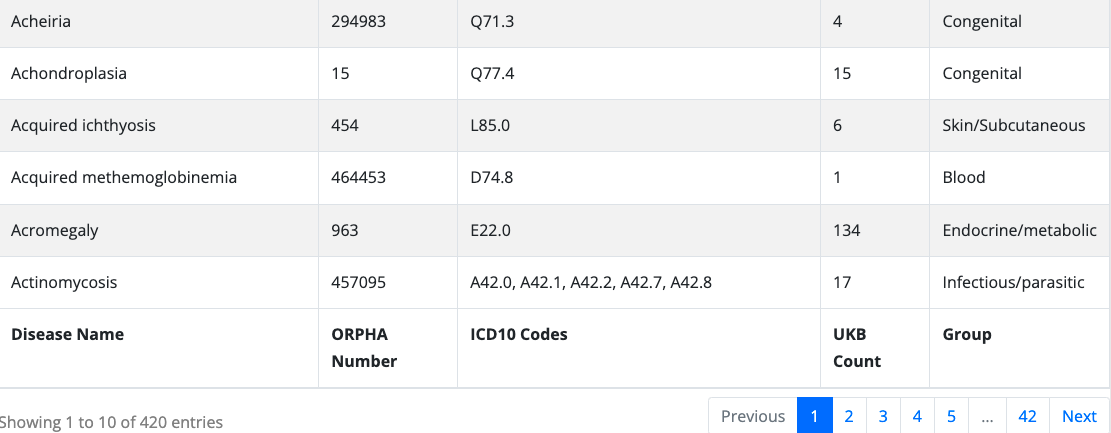


1. When hovering over a rare disease in the table, its background color will change to a darker grey, indicating it is clickable. Then, when clicking on a disease in the table, its background color will become yellow and information about the disease will be displayed above.


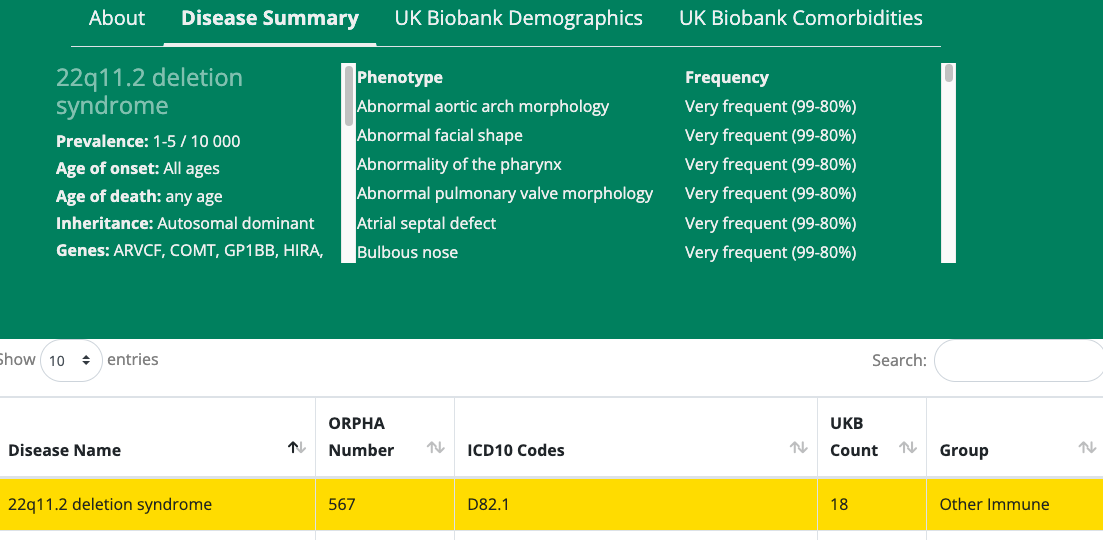


1. The first tab to be automatically selected at the top of the screen is “Disease Summary”. This shows information about the rare disease extracted and summarized from OrphaNet. On the left-hand side are specific details, including the disease prevalence, age of onset, age of death, inheritance mode, associated genes (according to OrphaNet) and disease categories, while on the right-hand side are the phenotypes known to occur with the disease, such as its symptoms. While this information is displayed in the most compact way possible, it is straightforward to access further information on the rare disease by clicking on its name in the left-hand side (again, when hovered over, it will change color, indicating it is clickable). Clicking on the name of the rare disease will open a new tab containing the OrphaNet website for the rare disease.
2. Clicking on ‘UK Biobank Demographics’ will navigate to the next tab, showing the age at recruitment and sex of participants for the rare disease, from our analysis of the UK Biobank. Both data points are shown in the context of the general population in the UK Biobank. The left plot shows the age distribution for the general population in the UK Biobank as a black line, while the mean age for the rare disease is indicated by a red line (with text overlaid in red to show the mean age of recruitment for the rare disease). The right plot shows the sex proportion for the general population in the UK Biobank using blue bars while the sex proportion for the rare disease is shown using red bars. So as to abide by the privacy restrictions of the UK Biobank, no age/sex information is shown for diseases with fewer than 5 individuals.


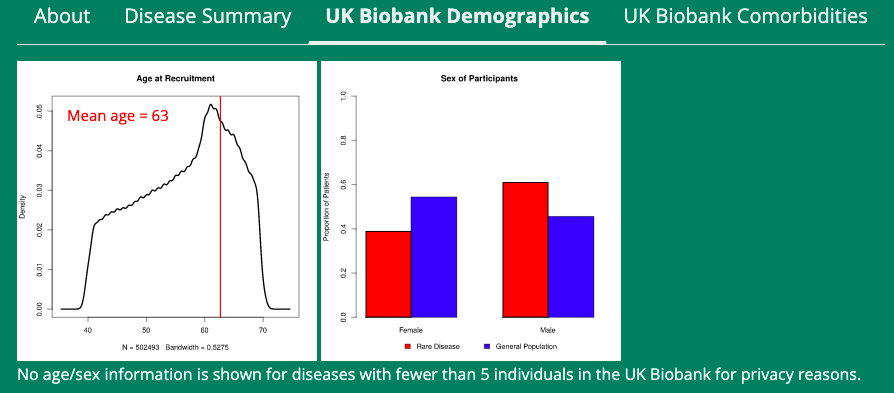


1. Clicking on ‘UK Biobank Comorbidities’ will navigate to the final tab. This shows a plot of the proportion of participants with different comorbidity groups (defined according to the ICD-10 chapters) compared to the general population in the UK Biobank. The proportions for the general population are shown in blue, while the proportions for the rare disease are shown in red. Along the y-axis is the proportion of participants (either from the background population or the specific disease) that have at least one comorbidity from each group. Again, so as to abide by the privacy restrictions of the UK Biobank, no age/sex information is shown for diseases with fewer than 5 individuals.


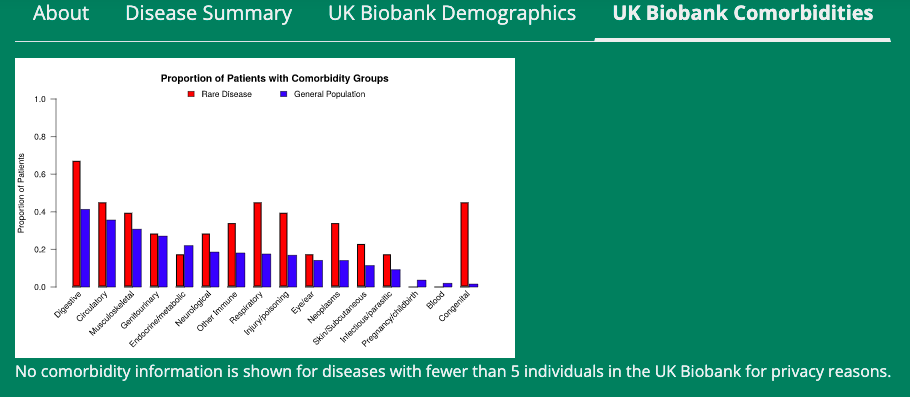


If you have any questions or comments regarding the website, please do not hesitate to contact Matthew Patrick (**mattpat@med.umich.edu**).
